# Chronic nonbacterial osteomyelitis — clinical and magnetic resonance imaging features

**DOI:** 10.1007/s00247-020-04827-6

**Published:** 2020-10-09

**Authors:** Paola d’Angelo, Laura Tanturri de Horatio, Paolo Toma, Lil-Sofie Ording Müller, Derk Avenarius, Elisabeth von Brandis, Pia Zadig, Ines Casazza, Manuela Pardeo, Denise Pires-Marafon, Martina Capponi, Antonella Insalaco, Benedetti Fabrizio, Karen Rosendahl

**Affiliations:** 1 Department of Radiology, Ospedale Pediatrico Bambino Gesu, Rome, Italy; 2 Section of Pediatric Radiology, Oslo University Hospital, Oslo, Norway; 3 Section of Pediatric Radiology, University Hospital of North Norway, 9019 Tromsø, Norway; 4 Department of Rheumatology, Ospedale Pediatrico Bambino Gesu, Rome, Italy; 5 Department of Clinical Medicine, UiT the Arctic University of Norway, Tromsø, Norway

**Keywords:** Bones, Children, Chronic nonbacterial osteomyelitis (CNO), Inflammation, Magnetic resonance imaging, Scoring system, Whole body

## Abstract

**Background:**

Chronic nonbacterial osteomyelitis (CNO) is a rare autoinflammatory bone disorder. Little information exists on the use of imaging techniques in CNO.

**Materials and methods:**

We retrospectively reviewed clinical and MRI findings in children diagnosed with CNO between 2012 and 2018. Criteria for CNO included unifocal or multifocal inflammatory bone lesions, symptom duration >6 weeks and exclusion of infections and malignancy. All children had an MRI (1.5 tesla) performed at the time of diagnosis; 68 of these examinations were whole-body MRIs including coronal short tau inversion recovery sequences, with additional sequences in equivocal cases.

**Results:**

We included 75 children (26 boys, or 34.7%), with mean age 10.5 years (range 0–17 years) at diagnosis. Median time from disease onset to diagnosis was 4 months (range 1.5–72.0 months). Fifty-nine of the 75 (78.7%) children presented with pain, with or without swelling or fever, and 17 (22.7%) presented with back pain alone. Inflammatory markers were raised in 46/75 (61.3%) children. Fifty-four of 75 (72%) had a bone biopsy. Whole-body MRI revealed a median number of 6 involved sites (range 1–27). Five children (6.7%) had unifocal disease. The most commonly affected bones were femur in 46 (61.3%) children, tibia in 48 (64.0%), pelvis in 29 (38.7%) and spine in 20 (26.7%). Except for involvement of the fibula and spine, no statistically significant differences were seen according to gender.

**Conclusion:**

Nearly one-fourth of the children presented with isolated back pain, particularly girls. The most common sites of disease were the femur, tibia and pelvic bones. Increased inflammatory markers seem to predict the number of MRI sites involved.

## Introduction

Chronic nonbacterial osteomyelitis (CNO) is an inflammatory, non-infectious disorder of the musculoskeletal system covering a wide clinical spectrum, with asymptomatic involvement of a single site at the one end and episodes of chronic recurrent multifocal osteomyelitis (CRMO) at the other end [1–4]. It is characterized by localized pain — often at night — and swelling, and primarily affects the metaphyses of long bones, although lesions can occur in any part of the skeleton [5–7]. Affection of tissues other than bone, such as skin, eyes, gastrointestinal tract and lungs, has been described [8]. CNO has a protracted course with numerous exacerbations and relapses at new and old sites. It primarily occurs in children and adolescents, with a peak of onset at 7–12 years and a reported incidence of 0.4–10.0/100,000 [1, 9, 10]. The true incidence is, however, thought to be higher because the findings are nonspecific. Although children with CNO frequently have mild to moderately increased levels of inflammatory markers, no findings — be they biochemical or imaging-based — are diagnostic for the disease. Currently, CNO is a diagnosis of exclusion, based on imaging or through histological examination of bone biopsies, which reveal acute and chronic inflammatory as well as reparative bone features like hyperostosis, without an infectious agent [11, 12].

Treatment strategies for children with CNO vary widely [13, 14]. Nonsteroidal anti-inflammatory drugs (NSAIDs) are commonly used as a first-line treatment, while second-line therapies include glucocorticoids, methotrexate, sulfasalazine, tumor necrosis factor (TNF)-α inhibitors and bisphosphonates [1, 7]. Although MRI, including whole-body examination, is widely used to diagnose and monitor CNO, little information exists on the use of imaging techniques, and standardized and validated assessment systems are lacking. Moreover, normal population-based standards for the MR appearances of the pediatric skeleton across age groups are nearly non-existent. Therefore, we combined clinical, laboratory (including histology when available) and imaging data from a large number of children with CNO to examine the patient characteristics, clinical presentation and pattern of involvement, with a special focus on MRI findings.

## Materials and methods

This multicenter retrospective study included children residing in Rome or Bergen with a diagnosis of CNO. We obtained data on age, gender, age of symptom onset, age at diagnosis, duration, clinical symptoms, laboratory and radiologic findings at diagnosis and follow-up of patients ages 0–18 years from the clinical journal systems and the radiology information system (RIS) and picture archiving and communication system (PACS) at Ospedale Pediatrico Bambino Gesu Hospital (OPBG), Rome, and Haukeland University Hospital (HUS), Bergen, respectively. The institutional board at OPBG approved the study.

### Patients

We included all children with mono- or multifocal inflammatory lesions diagnosed as CNO at the two participating pediatric centers during the period 2012–2018. The criteria for CNO were mono- or multifocal inflammatory bone lesions, duration of symptoms >6 weeks, and exclusion of infections and malignancy [7]. Demographic, clinical and laboratory data and histological findings, when available, were collected from the medical records. Normal ranges used for laboratory data were as follows: C-reactive protein (CRP)<0.5 mg/dL, erythrocyte sedimentation rate (ESR)<15 mm/h. In addition, we grouped inflammation (based on blood markers, ESR and CRP) as mild when at least one marker was raised but not >100, or moderate when at least one marker was >100.

### Imaging

**Fig. 1:**
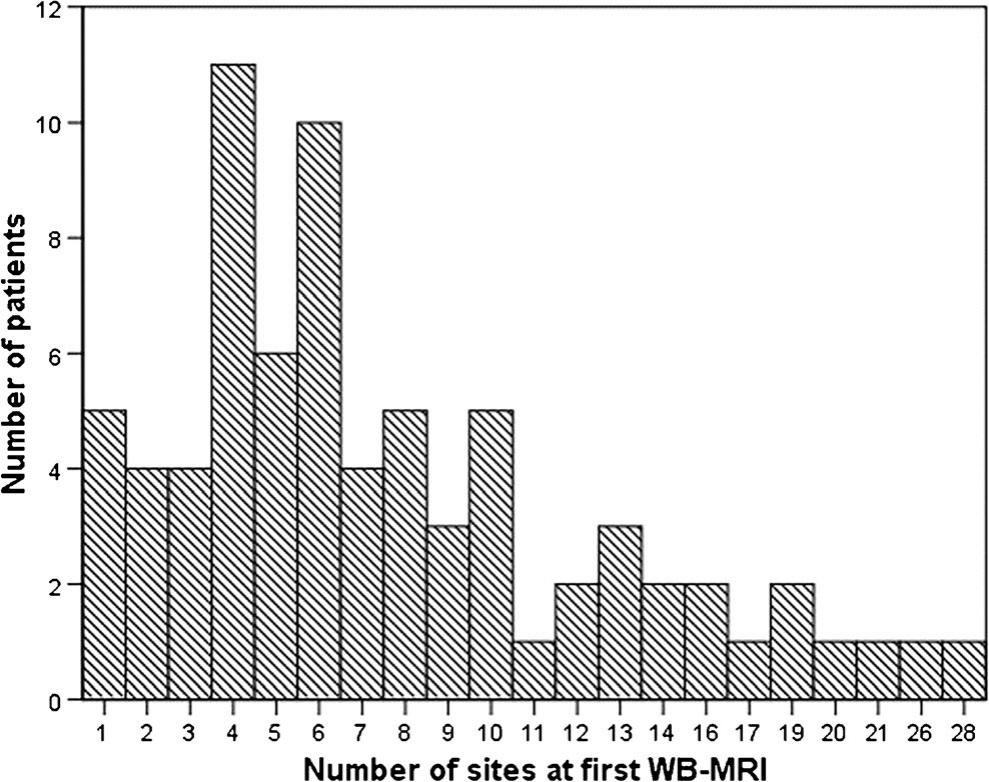
Graph shows number of sites based on the initial whole-body MRI (WB-MRI) examination in 67 of the 75 children with chronic nonbacterial osteomyelitis

**Fig. 2:**
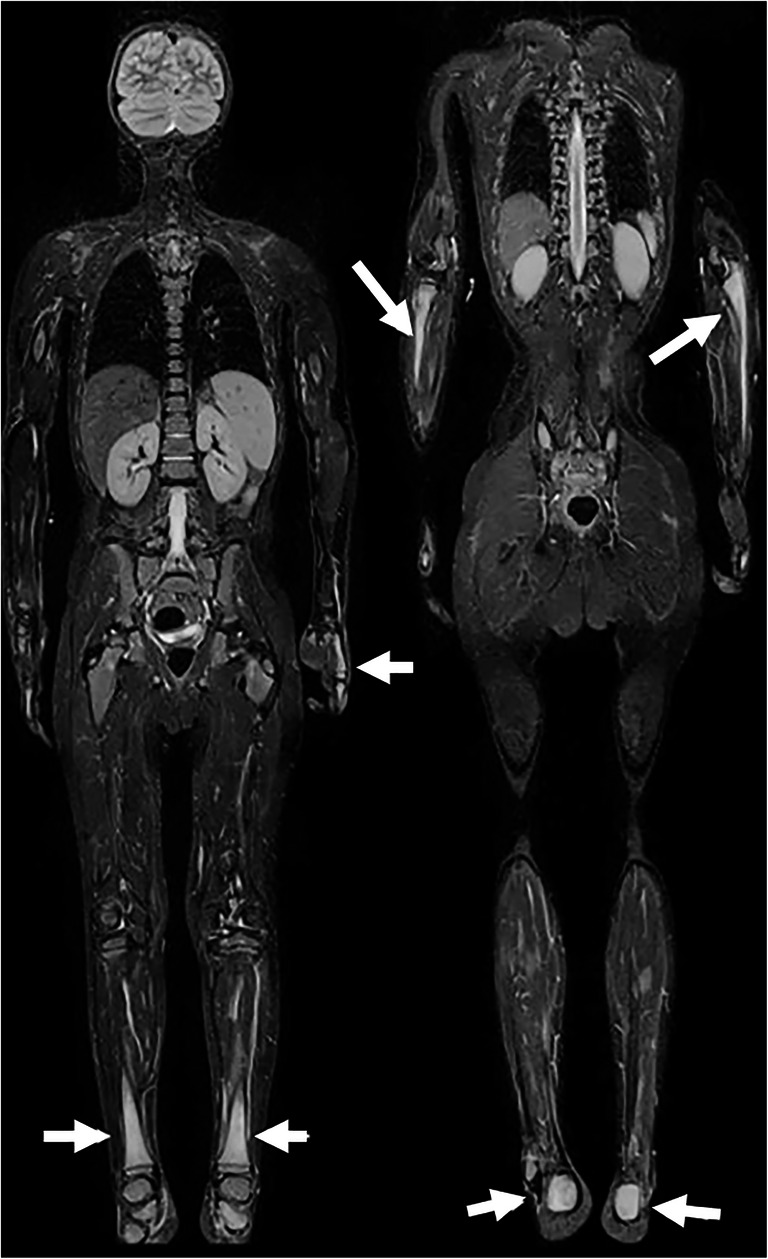
Chronic nonbacterial osteomyelitis in a 14-year-old boy. Whole-body coronal T2-W short tau inversion recovery images (left to right: anterior to posterior, repetition time/echo time = 5,000/58 ms) show high signal of both proximal ulnae, left hand, both lower legs and calcanei (*arrows*). Note normal signal from the proximal femurs

**Fig. 3:**
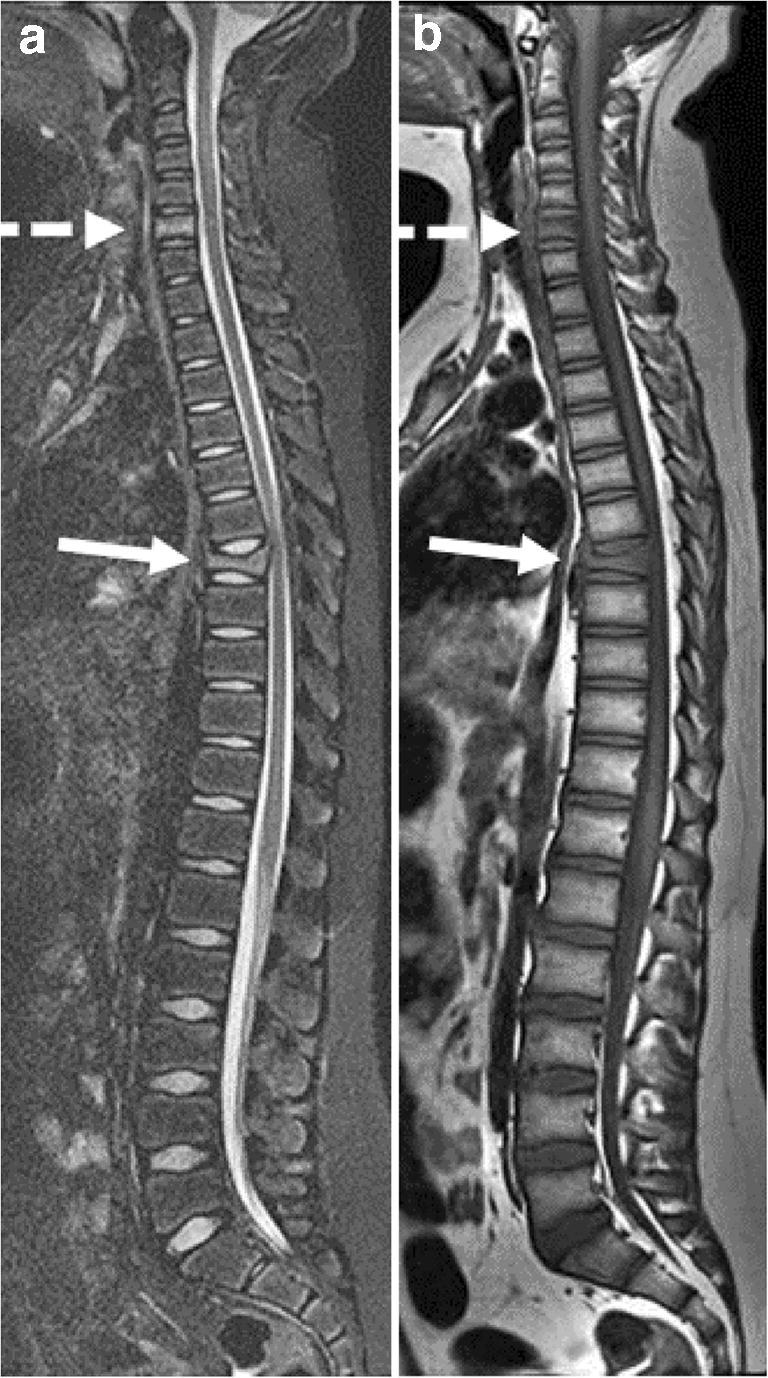
Chronic nonbacterial osteomyelitis in a 13-year-old boy. **a, b** Sagittal T2-W short tau inversion recovery MR image (repetition time/echo time [TR/TE] 5,000/58 ms) (**a**) and T1-W MR image (TR/TE 400/7 ms) (**b**) of the spine show signal change and vertebral compression of the 7th thoracic vertebrae (*solid arrow*) and signal changes of the 6th cervical vertebrae (*dashed arrow*)

**Fig. 4:**
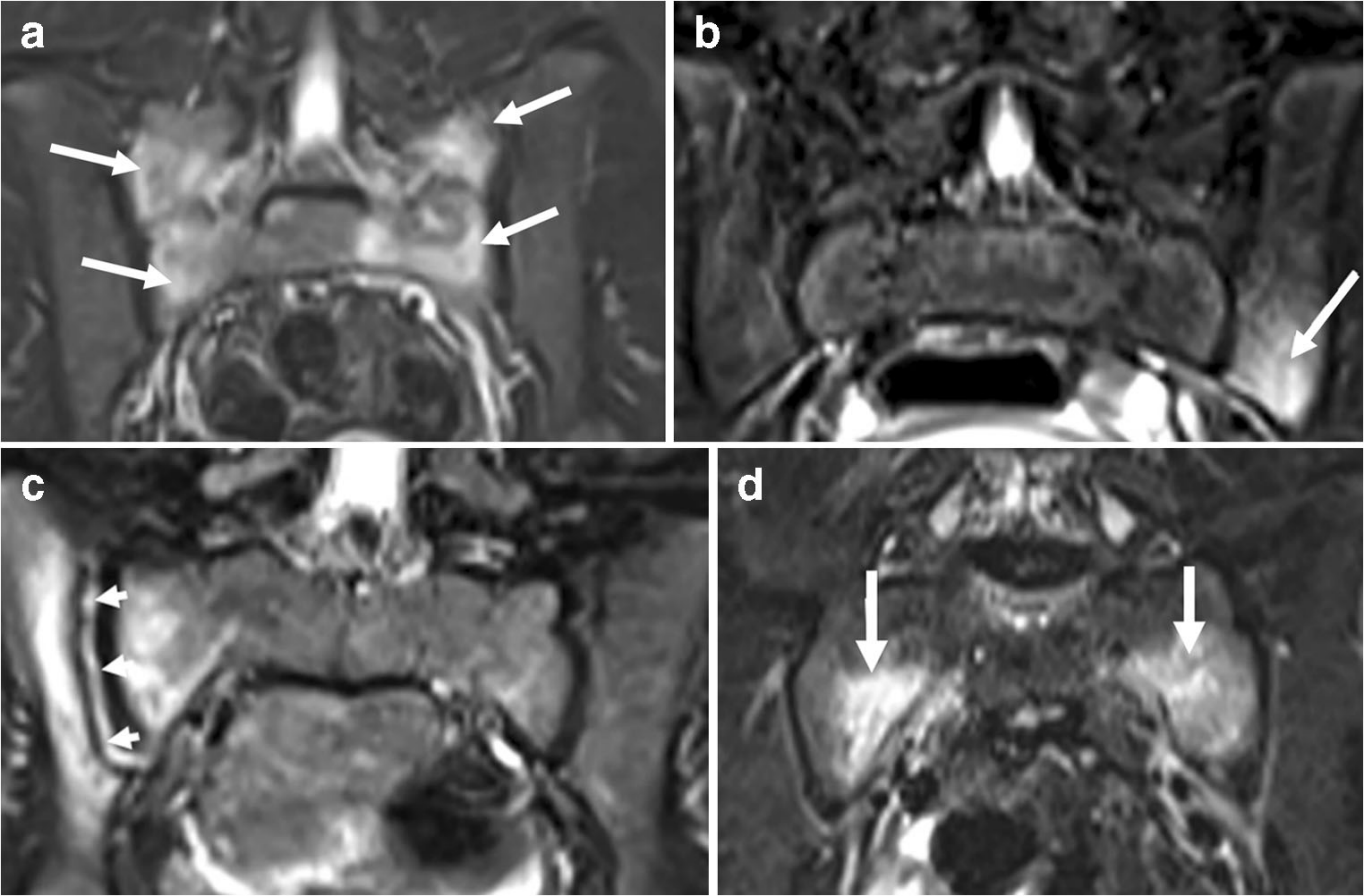
Chronic nonbacterial osteomyelitis with sacroiliac joint involvement in four children aged 10–15 years. Coronal T2-W short tau inversion recovery MR images (repetition time/echo time = 5,000/58 ms). **a** Bone marrow edema at the sacral side, bilaterally (*arrows*) in an 11-year-old girl. **b** Bone marrow edema at the left iliac side (*arrow*) in a 9-year-old girl. **c** High signal in the right joint space *arrows*, with surrounding bone marrow edema at the iliac and sacral sides in an 8-year-old girl. **d** Bone marrow edema at the sacral sides, bilaterally in a 15-year-old girl (*arrows*)

**Fig. 5:**
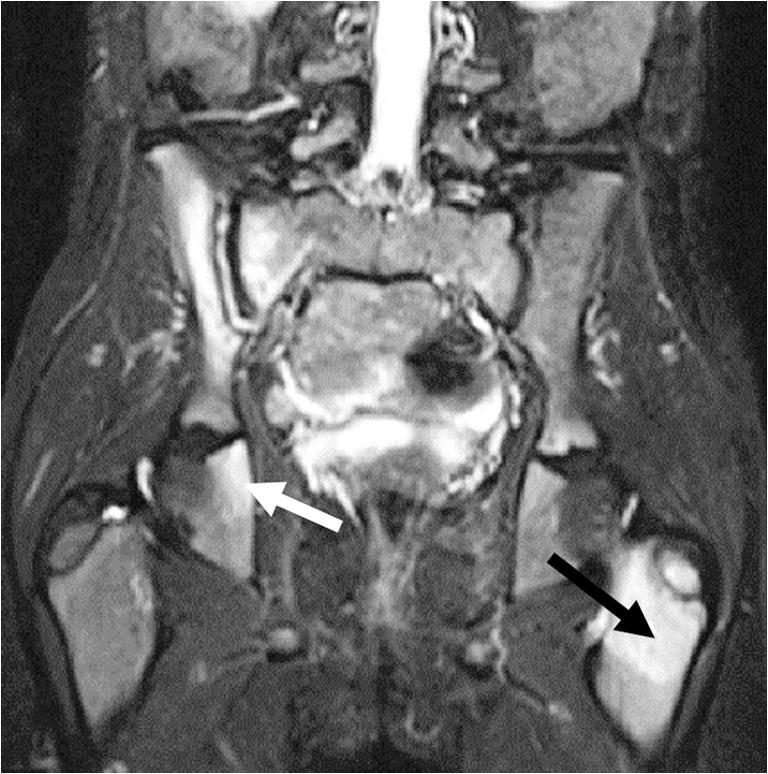
Chronic nonbacterial osteomyelitis in a 7-year-old girl. Coronal T2-W short tau inversion recovery MR image (repetition time/echo time = 5,000/58 ms) shows involvement of the right sacroiliac joint (high signal in the joint space with surrounding bone marrow edema), the right ischial bone (*white arrow*) and the left femoral metaphysis/apophysis (*black arrow*). Note the subtle high signal of the right femoral metaphysis, within normal variation

**Fig. 6:**
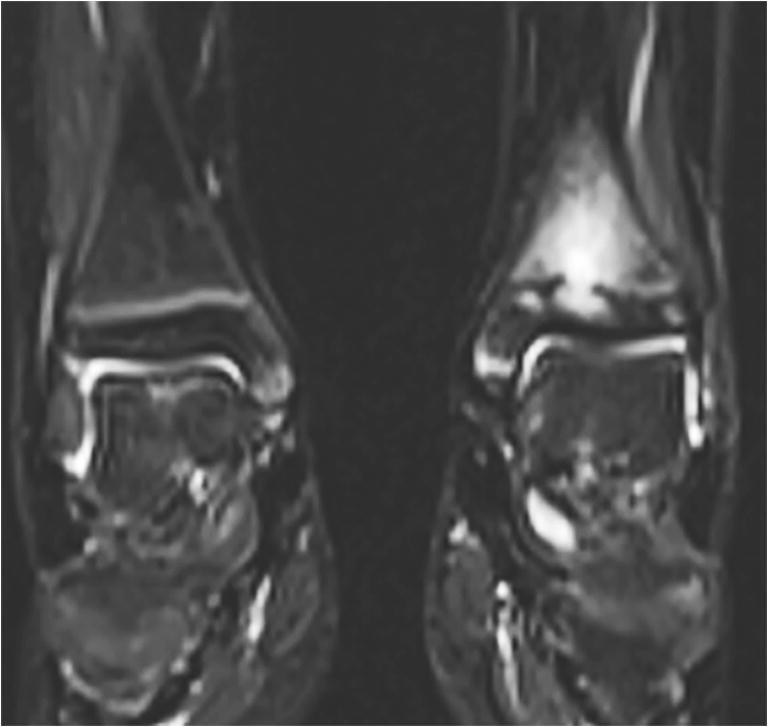
Chronic nonbacterial osteomyelitis in a 9-year-old girl. Coronal T2-W short tau inversion recovery MR image (repetition time/echo time = 5,000/58 ms) of both ankles shows involvement of the left metaphysis, growth plate and epiphysis. The right side is normal

**Fig. 7:**
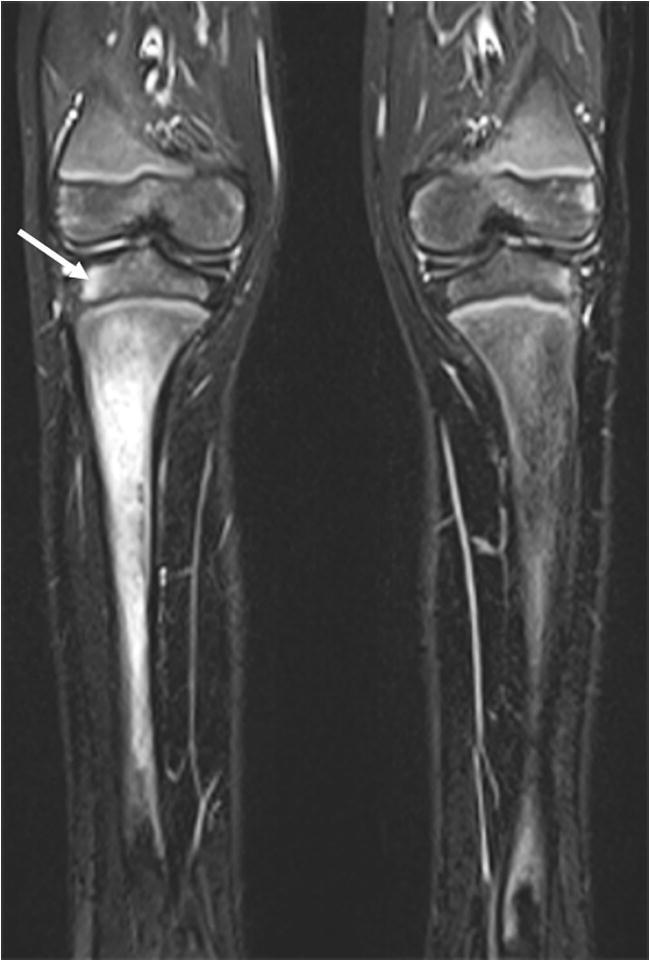
Chronic nonbacterial osteomyelitis in an 8-year-old boy. Coronal T2-W short tau inversion recovery MR image (repetition time/echo time = 5,000/58 ms) of the knees/legs shows involvement of the right tibial metaphysis and diaphysis, and the right epiphysis (*arrow*). The findings were confirmed on T1-weighted images

All MRIs performed at the time of diagnosis were re-analyzed by three of the authors (P.d'A., L.T.d.H. and K.R., with 3, 12 and 30 years of experience in paediatric radiology, respectively) (Fig. 1). We scored the following features: the bone(s) involved (noting epiphyseal, metaphyseal or diaphyseal location in the long bones), periosteal reaction, growth plate involvement, vertebral compression and the presence of soft-tissue inflammation (Figs. 2, 3, 4, 5, 6 and 7). Bone marrow edema and soft-tissue inflammation were defined as increased signal intensity (as compared to the remainder of the bone, or to the contralateral side) on water-sensitive sequences. All MRIs were performed on a 1.5-tesla (T) Siemens MRI system — at OPBG an Aera and at HUS an Avanto machine was used (Siemens, Erlangen, Germany). The whole-body MRI included a coronal short tau inversion recovery (STIR) sequence (repetition time/echo time/inversion time [TR/TE/TI] 5,000/58/160 ms and flip angle 147°; and a sagittal STIR sequence of the spine and feet, with additional T1-weighted images (TR/TE 400/7 ms) in equivocal cases. Typical duration of examination for the whole-body MRI was 35–45 min.

### Statistical analysis

Descriptive statistics were reported as mean (with standard deviations) and percentages or median (ranges). Differences between genders, as have been reported by others, were examined using *t*-tests or Fisher exact/chi-square tests as appropriate. Multiple linear regression analysis was used to test whether rise in inflammatory markers (ESR/CRP), time to diagnosis, age or gender significantly predicted the total number of bony sites involved at diagnosis. Statistical analyses were performed using SPSS version 25 (IBM, Armonk, NY). All tests were two-sided and statistical significance was set to *P*<0.05.

## Results

We included 75 children (26 boys, 34.7%) with a mean age of 10.5 years (standard deviation [SD] 3.2 years, range 0–17 years) at diagnosis. One child was younger than 2 years at time of diagnosis. The median time of symptom onset to diagnosis was 4 months (range 1.5 to 72.0 months). Fifty-nine of the 75 children (78.7%) presented with pain, with or without swelling or fever (Table 1). Seventeen children (22.7%) presented with isolated low back pain (spine or sacroiliac joints). Arthritis was seen in one child, who had ankle involvement. Cutaneous involvement, such as psoriasis, lupus erythematosus, papular lesions or pustulosis, was seen in 8 children (10.7%). Inflammatory markers were increased in 46/75 (61.3%) (Table 1). A biopsy of the most prominent bone lesion on imaging was performed in 54/75 children (72.0%); biopsies showed features consistent with subacute/chronic osteomyelitis in 27 and nonspecific changes in 17, and were inconclusive in the remaining 10. Five children (6.7%) presented with single-site involvement (two lower bones, one humerus, one spine and one mandible); all except one underwent bone biopsy.

**Table 1:** Age and symptoms for 75 children (26 boys) diagnosed to have chronic nonbacterial osteomyelitis (CNO) based on history and clinical, laboratory and imaging findings, with an additional biopsy in 54 children (72%)

	Boys (*n*=26)	Girls (*n*=49)	*P*-value^a^	Total (*n*=75)^b^
**Age at diagnosis, year, mean (SD)**	10.4 (3.3)	10.5 (3.1)	0.832	10.5 (3.2)
**Age at onset, years, mean (SD)**	9.5 (3.4)	10.0 (3.0)	0.534	9.8 (3.1)
**Symptoms at presentation, number (%)**				
Body pain	19	40	0.393	59 (78.7)
Localized pain^c^	9	10		19 (25.3)
Low back pain	3	14		17 (22.7)
Multifocal pain	5	5		10 (13.3)
Localized pain and swelling	1	11		12 (16.0)
Localized pain and fever (>38°C)	1	0		1 (1.3)
Fever only	2	2		4 (5.3)
Swelling only	1	2		3 (4.0)
Limp	3	5		8 (10.7)
**Elevated inflammatory blood makers**^**d**^**, number (%)**	13	33	0.209	46 (61.3)
Mild	10	20		30 (40.0)
Moderate	3	13		16 (21.3)
**Increased white blood count (WBC)**	0	1		1 (1.3)

^a^Differences between genders were examined using *t*-tests or chi-square tests as appropriate; 2-sided *P*-values are given; *P*<0.05 is significant

^b^One boy age 4 months presented with agitation, crying

^c^Other than low back pain

^d^Erythrocyte sedimentation rate and C-reactive protein

*SD* standard deviation

Magnetic resonance imaging was performed at baseline in all children; 68 had a whole-body MRI, revealing a median number of 6 sites (range 1–27) (Fig. 1). The long bones in the lower limbs were affected in 58/75 (77.3%) of the cases, the pelvis in 29/75 (38.7%), the spine in 20/75 (26.7%) and the long bones of the upper limbs in 20/75 (26.7%). Except for the fibula and spine, no statistically significant differences in involvement were seen according to gender (Table 2). A periosteal reaction was seen in four children, all of whom presented with focal pain, while soft-tissue involvement was seen in eight children.

**Table 2:** Number of children with bone changes at different sites consistent with inflammatory change as diagnosed on whole-body MRI at presentation (7 of the 75 children did not have a complete whole-body MRI)

	Boys (*n*=26)	Girls (*n*=49)	*P-*value^a^	Total (%) (*n*=75)
Lower limbs				
Femur	19	27	0.128	46 (61.3)
Tibia	18	30	0.492	48 (64.0)
Fibula	12	9	**0.011**	21 (28.0)
Feet	13	16	0.142	29 (38.7)
Upper limbs				
Humerus	4	11	0.467	15 (20.0)
Radius	3	6	0.929	9 (12.0)
Ulna	2	3	0.795	5 (6.7)
Hands	0	4	0.134	4 (5.3)
Spine	3	17	**0.031**	20 (26.7)
Sacrum	6	12	0.892	18 (24.0)
Sacroiliac joints	1	7	0.163	8 (10.7)
Pelvis	8	21	0.306	29 (38.7)
Ileum	4	15	0.149	19 (25.3)
Sternum	4	10	0.595	14 (18.7)
Scapula	2	6	0.543	8 (10.7)
Clavicle	3	11	0.248	14 (18.7)
Mandible	3	5	0.859	8 (10.7)
Ribs	3	1	0.081	4 (5.3)

^a^Differences between genders were examined using chi-square tests; 2-sided *P*-values are given; *P*<0.05 is significant (bold values)

Among the 68 children who had whole-body MRI, 82 epiphyseal, 80 metaphyseal and 5 physeal lesions were found in the long tubular bones. All physeal lesions were associated with metaphyseal or epiphyseal involvement. Twenty-seven children had diaphyseal lesions, all of which were located in the long bones of the lower extremities. Three of these 27 children had additional periosteal and soft-tissue reaction. Involvement of the epiphysis, metaphysis and diaphysis of the long tubular bones, by gender, is presented in Table 3.

**Table 3:** Number of children with epiphyseal, metaphyseal or diaphyseal involvement of at least one of the long bones, in 75 children with chronic nonbacterial osteomyelitis (7 of the 75 children did not have a complete whole-body MRI)

	Males (*n*=26)	Females (*n*=49)	*P-*value^a^	Total (%) (*n*=75)
Distal epiphysis	11	15	0.189	26 (34.7)
Proximal epiphysis	12	7	**0.027**	19 (25.3)
Diaphysis	12	15	0.131	27 (36.0)
Distal metaphysis	13	24	0.938	37 (49.3)
Proximal metaphysis	16	27	0.799	43 (57.3)

^a^Differences between genders were examined using chi-square tests; 2-sided *P*-values are given; *P*<0.05 is significant (bold values)

We used multiple linear regression analysis to test whether rise in inflammatory markers (ESR/CRP), time to diagnosis, age or gender significantly predicted the total number of bony sites involved at diagnosis. Preliminary analysis did not reveal any violation of the assumptions of normality, linearity, multicolinearity or homoscedasticity. The results of the regression indicated that the four predictors explained only 11.2% of the variance (R^2^=0.11, F (1.692), *P*=0.14). Elevation of inflammatory markers significantly predicted the number of sites (β=0.250, *P*=0.045). Neither age (β=0.186, *P*=0.132) nor gender (β=0.103, *P*=0.387) nor disease duration (β=0.025, *P*=0.841) predicted the number of involved bone sites significantly.

## Discussion

We have shown, in a large two-center cohort of children and adolescents diagnosed with CNO, that bony pain, with or without swelling, was the most common presenting symptom, that two-thirds were girls and that the femur, tibia and pelvis were most often involved. On whole-body MRI, epiphyses and metaphyses appeared to be equally involved. In contrast to previous reports, median time from time of onset to diagnosis was relatively low, with a median of 4 months, reflecting an increased awareness of the diagnosis during the last few years [7, 15]. Nearly one-fourth of the children presented with low back pain, particularly girls, with MR features exhibiting those of vertebral bone marrow edema. Age at presentation and gender distribution confirmed previous reports [7, 15].

The number of sites, both with and without symptoms at presentation, as demonstrated on the initial whole-body MRI varied between 1 and 27, with a median of 6. Whether the asymptomatic sites are true so-called silent lesions or subclinical disease, as suggested by others [1, 15], or merely reflect signal changes during normal bone growth remains unclear. From previous work we know that more than half of healthy children ages 5–16 years have bone-marrow-edema-like changes in the hand skeleton [16, 17]. Others have shown similar findings to the feet [18] and pelvis [19]. Further studies with a meticulous focus on the association between MRI findings and clinical symptoms are warranted to clarify the significance of asymptomatic MRI findings.

In a large registry study from 2018 including 486 children with CRMO from 19 countries, mean age 9.9 years, MRI revealed 4.1 sites per patient, as compared to 6 in our case series [7]. The relatively low number of whole-body MRI in their study, i.e. <30% of the patients, might explain part of the difference because whole-body MRI most likely introduces false-positive findings, or lesions. Population differences might also have played a role. Another study, by Andronikou et al. [20], which included 37 people with CRMO, reported on 8.6 lesions per patient based on whole-body MRI, with 89% of patients having multifocal disease. They noticed two patterns, namely a multifocal predominantly tibial involvement and pauci-focal clavicular and spinal disease. In our series we were not able to identify specific patterns in distribution.

The number of radiologic lesions has been used as a marker of disease activity in the context of the Pediatric CNO (PedCNO) score [21]. In addition to the number of radiologic lesions, the PedCNO includes ESR, severity of disease as judged by the physician, severity of the disease estimated by child or parent, and the Childhood Health Assessment Questionnaire (CHAQ) score. Zhao and colleagues [22] further described the characteristics of CNO lesions based on MRI findings using a grading system to score the severity of bone edema and soft-tissue inflammation as well as the presence of periosteal reaction, hyperostosis, growth plate damage and vertebral compression. Applying this scoring tool to a retrospective cohort of 18 people with CNO, the authors reported a significant decrease in the number of non-vertebral lesions and the maximum severity of bone edema in the group receiving aggressive treatment [14, 22]. They noted, however, that it remains unclear which MRI characteristics can be reliably assessed and to which degree they are sensitive to change. Moreover, it is not known how the MRI findings relate to other clinical assessment tools. In our large case series of 75 children with CNO, only 4 had a periosteal reaction, 3 related to diaphyseal involvement and 1 in a rib. Thus, periosteal reaction is probably too rare to be used as prognostic support, as are soft-tissue and physeal involvement. As for the extent and intensity of bone marrow edema, we did not score this in particular. However, being a key finding in CNO, it is reasonable to believe that the extent/degree of bone edema might be of value, assuming that we will be able to distinguish between true inflammatory change and changes caused by normal growth. Currently, there is no agreement on a standardized evaluation tool [14].

In our series, elevation of the inflammatory markers significantly predicted the number of MRI sites, suggesting that the number of MRI sites represents a marker for disease activity.

There are several limitations to our study, including its retrospective design and the lack of whole-body MRI in seven of the patients. Moreover, image quality is suboptimal for assessing physeal lesions and periosteal reaction, thus these features might be underestimated. The possibility of artifacts, particularly for borderline structures such as the sternum and ribs, is also a potential bias. The strengths of the study were the large number of children with whole-body MRI and the meticulous image analysis.

## Conclusion

Nearly one-fourth of children with CNO, particularly girls, presented with isolated back pain only. The most common sites of disease were the femur, tibia and pelvic bones. Elevation of inflammatory markers seems to predict the number of MRI sites of disease.

## References

[1] Hofmann SR, Schnabel A, Rosen-Wolff A (2016). Chronic nonbacterial osteomyelitis: pathophysiological concepts and current treatment strategies. J Rheumatol.

[2] Girschick HJ, Raab P, Surbaum S (2005). Chronic non-bacterial osteomyelitis in children. Ann Rheum Dis.

[3] Falip C, Alison M, Boutry N (2013). Chronic recurrent multifocal osteomyelitis (CRMO): a longitudinal case series review. Pediatr Radiol.

[4] Iyer RS, Thapa MM, Chew FS (2011). Chronic recurrent multifocal osteomyelitis: review. AJR Am J Roentgenol.

[5] Grote V, Silier CC, Voit AM, Jansson AF (2017) Bacterial osteomyelitis or nonbacterial osteitis in children: a study involving the German Surveillance Unit for Rare Diseases in Childhood. Pediatr Infect Dis J 36:451–456 10.1097/INF.0000000000001469 28403046

[6] Silier CCG, Greschik J, Gesell S (2017). Chronic non-bacterial osteitis from the patient perspective: a health services research through data collected from patient conferences. BMJ Open.

[7] Girschick H, Finetti M, Orlando F (2018). The multifaceted presentation of chronic recurrent multifocal osteomyelitis: a series of 486 cases from the Eurofever international registry. Rheumatology.

[8] Costa-Reis P, Sullivan KE (2013). Chronic recurrent multifocal osteomyelitis. J Clin Immunol.

[9] Taddio A, Zennaro F, Pastore S, Cimaz R (2017) An update on the pathogenesis and treatment of chronic recurrent multifocal osteomyelitis in children. Paediatr Drugs 19:165–172 10.1007/s40272-017-0226-4 28401420

[10] Berkowitz YJ, Greenwood SJ, Cribb G (2018). Complete resolution and remodeling of chronic recurrent multifocal osteomyelitis on MRI and radiographs. Skelet Radiol.

[11] Jurik AG, Moller BN (1986). Inflammatory hyperostosis and sclerosis of the clavicle. Skelet Radiol.

[12] Carr AJ, Cole WG, Roberton DM, Chow CW (1993) Chronic multifocal osteomyelitis. J Bone Joint Surg Br 75:582–591 10.1302/0301-620X.75B4.8331113 8331113

[13] Ramanan AV, Hampson LV, Lythgoe H (2019). Defining consensus opinion to develop randomised controlled trials in rare diseases using Bayesian design: an example of a proposed trial of adalimumab versus pamidronate for children with CNO/CRMO. PLoS One.

[14] Zhao Y, Wu EY, Oliver MS (2018). Consensus treatment plans for chronic nonbacterial osteomyelitis refractory to nonsteroidal antiinflammatory drugs and/or with active spinal lesions. Arthritis Care Res.

[15] Roderick MR, Shah R, Rogers V (2016). Chronic recurrent multifocal osteomyelitis (CRMO) — advancing the diagnosis. Pediatr Rheumatol Online J.

[16] Ording Müller L-S (2012) Establishment of normative MRI standards for the paediatric skeleton to better outline pathology. Focused on juvenile idiopathic arthritis. Doctoral dissertation. University of Tromsø, Tromsø

[17] Avenarius DFM, Ording Muller LS, Rosendahl K (2017). Joint fluid, bone marrow edemalike changes, and ganglion cysts in the pediatric wrist: features that may mimic pathologic abnormalities — follow-up of a healthy cohort. AJR Am J Roentgenol.

[18] Shabshin N, Schweitzer ME, Morrison WB (2006). High-signal T2 changes of the bone marrow of the foot and ankle in children: red marrow or traumatic changes?. Pediatr Radiol.

[19] Ording Muller LS, Avenarius D, Olsen OE (2011). High signal in bone marrow at diffusion-weighted imaging with body background suppression (DWIBS) in healthy children. Pediatr Radiol.

[20] Andronikou S, Mendes da Costa T, Hussien M, Ramanan AV (2019). Radiological diagnosis of chronic recurrent multifocal osteomyelitis using whole-body MRI-based lesion distribution patterns. Clin Radiol.

[21] Beck C, Morbach H, Beer M (2010). Chronic nonbacterial osteomyelitis in childhood: prospective follow-up during the first year of anti-inflammatory treatment. Arthritis Res Ther.

[22] Zhao Y, Chauvin NA, Jaramillo D, Burnham JM (2015). Aggressive therapy reduces disease activity without skeletal damage progression in chronic nonbacterial osteomyelitis. J Rheumatol.

